# Aggregated transfer factor of ^137^Cs in edible wild plants and its time dependence after the Fukushima Dai-ichi nuclear accident

**DOI:** 10.1038/s41598-022-09072-5

**Published:** 2022-03-25

**Authors:** Momo Takada, Tetsuo Yasutaka, Seiji Hayashi, Mai Takagi, Keiko Tagami

**Affiliations:** 1 National Institute of Advanced Industrial Science and Technology, 1-1-1 Higashi, Tsukuba, Ibaraki 305-8567 Japan; 2 National Institute for Environmental Studies, 10-2 Fukasaku, Miharu, Fukushima 963-7700 Japan; 3 National Institutes for Quantum Science and Technology, 4-9-1 Anagawa, Inage-ku, Chiba, 263-8555 Japan

**Keywords:** Forest ecology, Risk factors

## Abstract

We obtained the aggregated transfer factor (*T*_ag_) for 10 common edible wild plant species (four perennial spermatophytes, bamboo shoot, two tree species, and three perennial pteridophytes) in northeastern Japan. Measurement of *T*_ag_ was carried out in 2012–2019 and we also used publicly available data for 2012–2019: food monitoring data and total deposition data from an airborne survey. The *T*_ag_ obtained from actual measurements agreed well with *T*_ag_ values calculated from the publicly available data. The sampling locations were only identified at the municipal level and uncertainty of the deposition for the publicly available data, and thus *T*_ag_ values showed substantial variation. The *T*_ag_ of the perennial spermatophytes, including bamboo shoot, and perennial pteridophytes showed single exponential decline with effective half-lives of approximately 2 years, whereas those of tree species did not show distinct temporal change. These results imply that data since 2014 are applicable for *T*_ag_ estimation for long-term potential ingestion dose in the future to the public because of the slow decline. The calculated *T*_ag_ values of all species for 2014–2019 ranged from 6.1 × 10^−5^ to 5.2 × 10^−3^ m^2^/kg-fresh mass. The maximum *T*_ag_ value was observed for the tree koshiabura (*Chengiopanax sciadophylloides*) and the minimum value was observed for the perennial spermatophyte giant butterbur (*Petasites japonica*). Tree species showed higher *T*_ag_ than spermatophyte and pteridophyte perennials.

## Introduction

Enhanced radiocesium levels in wild foodstuffs in forests persist for longer time periods than in agricultural crops because of factors such as the small amounts of discharge and recycling within the ecosystem^1,2^. Although 10 years have passed since TEPCO’s Fukushima Dai-ichi nuclear power plant accident (hereafter, the Fukushima accident), the radiocesium concentrations in wild foodstuffs (e.g., wild mushrooms and edible wild plants) often exceed the Japanese limit of 100 Bq/kg for ^134^Cs and ^137^Cs on a fresh mass (FM) basis, whereas exceedances of this limit have rarely been reported for agricultural products since 2015^3^. Edible wild plants, comprising spermatophytes and pteridophytes from which new shoots are consumed as leaf vegetables, are the most familiar wild foods in Japan. The collection of edible wild plants in fields or mountains in spring is a tradition in Japanese culture. Estimation of the potential ingestion dose in the future to the public from the consumption of edible wild plants containing ^134^Cs and ^137^Cs is required from a radiation protection point of view. This estimation will improve the quality of life for local residents hoping to restore the enjoyment of eating wild foods^4^.

To evaluate the potential ingestion dose in the future to the public from consumption of edible wild plants, estimation of the radionuclide content in edible wild plants is required, for which use of the aggregated transfer factor (*T*_ag_) is appropriate. This is defined as the radionuclide activity concentration in edible parts (Bq/kg) divided by radionuclide deposition in the soil (Bq/m^2^). *T*_ag_ is commonly applied to estimate radionuclide concentrations in foodstuffs such as berries, mushrooms, and game harvested in natural or semi-natural ecosystems including upland areas or forests^5^. Previous studies have compiled *T*_ag_ data for trees, berries, mushrooms, and game animals^6–8^. The *T*_ag_ assumes equilibrium conditions in the environment by definition, but *T*_ag_ values usually change over time owing to soil fixation processes and radionuclide migration^2,9^. Few *T*_ag_ values of Japanese edible wild plants in an equilibrium condition from before the Fukushima accident are available, although decrease in the radiocesium concentration in several edible wild plants has been observed after the accident^10,11^ and, accordingly, the *T*_ag_ values decreased. To estimate the long-term potential ingestion dose attributable to consumption of edible wild plants, *T*_ag_ should be calculated using data that do not show large temporal change because high *T*_ag_ values soon after contamination cause an overestimation of the long-term ingestion dose. Tagami and Uchida^10^ examined the temporal change in radiocesium concentration for three perennial edible wild plants from July 2011 to May 2016 in Chiba Prefecture. The decline of the concentrations could be approximated by exponential functions with two components; effective half-lives representing the rapid and slow loss were 103–230 days and 970–3830 days, respectively. Based on these results, *T*_ag_ values for assessing long-term potential ingestion dose should be estimated from data since 2014. Tagami et al.^12^ observed no decreasing trend in radiocesium concentrations in five species (perennial and woody spermatophytes, and pteridophytes) since 2016 when analyzing monitoring results reported from local governments in Fukushima Prefecture, and thus *T*_ag_ values were calculated with data since 2016.

Following the Fukushima accident, although the *T*_ag_ of edible wild plants has been studied previously, the number of species analyzed to date remains limited. A far greater number of edible wild plant species are utilized in Japan and about 20 species of edible wild plants have been consumed, according to interview-based surveys of local residents in rural areas of northeastern Japan^13,14^. The *T*_ag_ for additional species is required to accurately estimate the potential ingestion dose to the public from consumption of edible wild plants. However, most edible wild plant species are not cultivated in agricultural fields, and finding them requires searching a wide area in fields and mountains on foot. Therefore, for many species, conducting a continuous field survey to directly determine *T*_ag_ values is difficult.

Food radioactivity has been monitored since the Fukushima accident by the Ministry of Health, Labour and Welfare (MHLW)^15^. The monitoring data are publicly available and include the radioactivity of various edible wild plants. These data may be applicable for calculation of *T*_ag_ for a variety of edible wild plant species. To calculate *T*_ag_, soil radioactivity data at each sampling location are required, but the MHLW food monitoring data are not accompanied by the corresponding soil data. We considered total radiocesium deposition data from an airborne survey by the Ministry of Education, Culture, Sports, Science and Technology (MEXT)^16^ to be appropriate to calculate *T*_ag_, instead of soil contamination data. The reason for this is that radiocesium was intercepted by the forest canopy soon after the accident, and then the radiocesium mostly transferred to the forest floor with time^17^. Kato et al. reported that soil radiocesium activity in June–July 2011 agreed well with values to the results of the third airborne monitoring survey of May–July, 2011^18^.

In the present study, we compared *T*_ag_ values of edible wild plants calculated using publicly available data, and those obtained with actual measured samples, to confirm the applicability of the publicly available monitoring data. To determine an appropriate period for estimating the potential long-term ingestion dose resulting from the intake of edible wild plants, we also calculated the effective half-life (*T*_eff_) from the publicly available data, taking into account the physical half-life and ecological half-life. The *T*_ag_ values for the edible wild plant species were calculated according to the *T*_eff._, which can be appropriate to estimate the long-term potential ingestion dose resulting from consumption of these edible wild plants.

## Methods

### Edible wild plants and soil samples for *T*_ag_ calculation

#### Sample collection

We collected edible wild plants and soil samples, and calculated *T*_ag_ for three different parts of two deciduous perennial spermatophytes [giant butterbur (limb and petiole of *Petasites japonicas*), butterbur scape (flower bud of *Petasites japonicas*), and udo (*Aralia cordata*)], two deciduous trees [fatsia sprout (*Aralia elata*) and koshiabura (*Chengiopanax sciadophylloides*)], and one deciduous pteridophyte perennial [western bracken fern (*Pteridium aquilinum*)]. The sampling locations were located on the campus of the National Institutes for Quantum and Radiological Science and Technology in Chiba Prefecture (about 220 km south of the Fukushima Dai-ichi Nuclear Power Plant) for giant butterbur and butterbur scape in 2012–2019, and in fields and mountains in Kawamata town and Iitate village, Fukushima Prefecture (about 40 km northwest of the power plant) for udo, fatsia sprout, koshiabura, and western bracken fern in 2018–2019. The names of the plants in the present study are based on traditional distinctions in the regions, not biological classification, and we identified the samples with local people in the collection areas. For the fields and mountains in Kawamata town and Iitate village, we obtained permission from the landowners to enter the fields for collection, and we worked with the residents to collect wild plants. Litter and soil samples were also collected separately at each sampling location for the edible wild plants, from 20 × 20-cm areas and with a core sampler (5 cm diameter and 5 cm depth). The Forestry Agency reported that 75–88% of soil radiocesium was observed in 0–5 cm layers in six forests in Fukushima prefecture in 2019^19^. In 10 undisturbed croplands in eastern Japan, about 70% of ^134^Cs in soil was kept in 0–5 cm layers in 2018^20^. Accordingly, we used ^137^Cs concentration data in soil 0–5 cm as soil deposition in the present study. Litter and soil samples were collected from 2–3 points at each sampling site.

#### Measurement

The collected samples of the edible wild plants were immediately transported to the laboratory and the fresh mass was recorded. All samples were oven dried at 80 °C, the dried weight was recorded, and the samples were crushed. Each sample was mixed well and enclosed in a 100 ml plastic container for radioactivity measurement. The litter and soil samples were also oven dried at 80 °C and enclosed in 100 ml plastic containers. The radioactivity of each sample was measured using a HPGe detector (Seiko EG&G, Japan). Radioactivity was measured for as long as 200,000 s, depending on the radioactivity of the samples. Radiocesium activity concentrations (Bq/kg-FM) of the edible wild plants were derived on a FM basis from the ratio between the fresh and dry masses. Deposition densities of radiocesium (Bq/m^2^) were combined for litter and soil samples because the litter layer still contains negligible amounts of radiocesium^17^.

### Publicly available data

#### Radiocesium activity in edible wild plants from food monitoring data

Data for edible wild plants were downloaded from the MHLW food monitoring data^15^ on 28 June, 2019. The data were recorded from both cultivated and wild material. We excluded data for cultivated plants from the present analysis. Twelve parts of 11 species of edible wild plants were included as more than 300 measurements were recorded in the period 2011–2019: five deciduous perennial spermatophytes [giant butterbur, butterbur scape, udo, uwabamisou (*Elatostema umbellatum*), momijigasa (*Parasenecio delphiniifolius*), and ohbagiboushi (*Hosta sieboldiana*)], bamboo shoot (*Phyllostachys* spp.), two deciduous trees (fatsia sprout and koshiabura), and three deciduous perennial pteridophytes [western bracken fern, ostrich fern (*Matteuccia struthiopteris*), and Japanese royal fern (*Osmunda japonica*)]. These 11 species are indicated to be common edible wild plants in the study region based on the large number of records. However, ohbagiboushi was excluded from the calculation because only 28 radioactivity detections were recorded in the total of ~ 500 measurements. Data for 2012–2019 were used for the present analysis because of the low number of measurements in 2011. As the season for collecting some edible wild plants starts in December and continues until July in the following year, the data for a specific year comprised those from December of the previous year to July of the current year. The monitoring data contained the sampling location at the municipal level, but more detailed location data were not identified. We selected the monitoring data collected at the municipalities where radiocesium deposition data were monitored. We calculated *T*_ag_ for the edible wild plants on a FM basis (m^2^/kg-FM) in the present study. Most *T*_ag_ estimates for forest products, such as trees, mushrooms, and berries, have been evaluated on a dry-weight basis in previous studies^6^ and in international literature^5,21^. However, the radioactivity concentrations of the present monitoring data were recorded on a FM basis (Bq/kg-FM), but the water content of the samples was not recorded.

#### Radiocesium deposition data from airborne survey

The total radiocesium deposition data were obtained from the results of the fifth airborne monitoring survey of 28 July, 2012 conducted by MEXT^16^. The measurement value is the average of measurements within a circle of about 600 m diameter under the aircraft, with a flight path width of 1.85 km within 80 km of the nuclear plant and 3 km outside the 80 km. Our obtained data comprised radiocesium (^134^Cs and ^137^Cs) deposition densities at the center points of the sampling squares (approximately 250 × 250 m). The results of only one monitoring event, on 28 July, 2012, were adapted for the entire period of the food monitoring data in the present study, although airborne monitoring surveys by MEXT were conducted several times. The residence half-time of the radiocesium in agricultural soil in Japan is approximately 15 years^22^, and radiocesium discharge into rivers from catchments was small after the Fukushima accident^23^. Thus, only one survey for deposition data could be used for food monitoring data in 2012–2019.

We used a representative deposition value for each municipality because sampling locations of the edible wild plants from the food monitoring data were determined at the municipal level. A geometric mean value of the deposition densities was used as the representative value for each municipality as the deposition densities in each municipality showed log-normal distributions rather normal distributions. For areas where the deposition density was less than 10,000 Bq/m^2^, we used the half value, 5000 Bq/m^2^, because the data indicated “< 10,000 Bq/m^2^” uniformly. The municipalities in which more than 30% of the area had a deposition density less than 10,000 Bq/m^2^ were excluded from the present analysis because of the large uncertainty in the deposition. The final area for the present analysis comprised 95 municipalities in seven prefectures (Iwate, Miyagi, Fukushima, Ibaraki, Tochigi, Gunma, and Chiba).

#### Data used for T_ag_ calculation

The data for the 11 parts of 10 species used for *T*_ag_ calculation are summarized in Table 1. All detected samples for the publicly available data were used in the *T*_ag_ calculation. The detected sample number accounted for 18–95% of the total number of measurements; for most samples, the reported results were less than the detection limit. Data less than the detection limit were excluded for *T*_ag_ calculation from publicly available data, which avoids underestimation of ingestion dose although there is the possibility of overestimation of *T*_ag_.

**Table 1:** Data used for calculation of the aggregated transfer factor of 11 parts of 10 edible wild plant species in 2012–2019.

Vernacular name	Scientific name	Plant category	Publicly available data^a^					Measured data^a^			
			No. of measurements	Percentage of measurements above the detection limit (%)	No. of prefectures/municipalities	Concentration range (Bq/kg-FM)	Corresponding soil deposition range (kBq/m^2^)	No. of measurements	Location and sampling period	Concentration range (Bq/kg-FM)	Corresponding soil deposition range (kBq/m^2^)
Giant butterbur	*Petasites japonicus*	Spermatophyte, deciduous perennial	707	18	7/63	ND-156	12.2–7.34 × 10^2^	44	QST^b^ (Chiba), 2012–2018	0.4–8.4	9.2–14.8
Butterbur scape	*Petasites japonicus*	Spermatophyte, deciduous perennial	536	29	5/59	ND-324	12.2–6.87 × 10^2^	22	QST (Chiba), 2012–2019	0.8–23.2	9.2–14.8
Udo	*Aralia cordata*	Spermatophyte, deciduous perennial	383	18	4/53	ND-332	14.0–7.34 × 10^2^	2	Kawamata^c^ (Fukushima), 2018–2019	6.8–41.6	33.9–54.7
Uwabamisou	*Elatostema umbellatum*	Spermatophyte, deciduous perennial	149	70	3/29	ND-435	12.6–1.51 × 10^2^				
Momijigasa	*Parasenecio delphiniifolius*	Spermatophyte, deciduous perennial	100	57	4/22	ND-151	12.2–1.21 × 10^2^				
Bamboo shoot	*Phyllostachys* spp.	Spermatophyte	3596	69	7/74	ND - 737	12.2–6.87 × 10^2^				
Fatsia sprout	*Aralia elata*	Spermatophyte, deciduous tree	501	49	7/62	ND-898	12.2–6.87 × 10^2^	3	Kawamata (Fukushima), 2019	29.6–2.25 × 10^2^	1.30 × 10^2^–2.87 × 10^2^
Koshiabura	*Chengiopanax sciadophylloides*	Spermatophyte, deciduous tree	85	95	6/56	ND-7.86 × 10^3^	12.2–6.87 × 10^2^	7	Kawamata, Iitate^d^ (Fukushima), 2018–2019	6.07 × 10^2^–1.43 × 10^4^	78.8–8.65 × 10^2^
Western bracken fern	*Pteridium aquilinum*	Pteridophyte, deciduous perennial	1109	38	7/61	ND-452	12.2–6.87 × 10^2^	2	Kawamata (Fukushima), 2018–2019	2.9–22.1	76.5–3.57 × 10^2^
Ostrich fern	*Matteuccia struthiopteris*	Pteridophyte, deciduous perennial	265	49	5/52	ND-412	12.2–6.87 × 10^2^				
Japanese royal fern	*Osmunda japonica*	Pteridophyte, deciduous perennial	73	64	6/28	ND-2.99 × 10^3^	14.0–6.87 × 10^2^				

^a^Radioactivity is for ^137^Cs and is decay-corrected to the day of sample collection.

^b^National Institutes for Quantum and Radiological Science and Technology (QST) in Chiba Prefecture, about 220 km south of the Fukushima Dai-ichi Nuclear Power Plant.

^c^Kawamata town in Fukushima Prefecture, about 40 km northwest of the power plant.

^d^Iitate village in Fukushima Prefecture, about 40 km northwest of the power plant.

### Analysis

We calculated the *T*_ag_ for ^137^Cs because ^137^Cs has a long half-life (*T*_1/2_ = 30 years) and therefore is suitable for long-term dose assessment. Radioactivity of ^137^Cs for all samples was decay-corrected to 11 March, 2011. The aggregated transfer factor (*T*_ag_) was calculated in accordance with IAEA-TRS 472^5^:

1 $$ T_{{{\text{ag}}}} \left( {{\text{m}}^{{2}} /{\text{kg}} - {\text{FM}}} \right) = {{^{{{137}}} {\text{Cs}}\,{\text{in}}\,{\text{edible}}\,{\text{ part}}\,\left( {{\text{Bq}}/{\text{kg}} - {\text{FM}}} \right)} \mathord{\left/ {\vphantom {{^{{{137}}} {\text{Cs}}\,{\text{in}}\,{\text{edible}}\,{\text{ part}}\,\left( {{\text{Bq}}/{\text{kg}} - {\text{FM}}} \right)} {^{{{137}}} {\text{Cs }}\,{\text{deposition}}\,\left( {{\text{Bq}}/{\text{m}}^{{2}} } \right)}}} \right. \kern-\nulldelimiterspace} {^{{{137}}} {\text{Cs }}\,{\text{deposition}}\,\left( {{\text{Bq}}/{\text{m}}^{{2}} } \right)}}. $$

To determine an appropriate period for estimation of long-term potential ingestion dose rate, we also calculated the effective half-life (*T*_eff_), which takes into account the physical half-life and ecological half-life. *T*_eff_ was determined using temporal change in ^137^Cs concentrations normalized by the deposition that is temporal change in *T*_ag_ value in the present study, which is defined as

2 $$ T_{{{\text{eff}}}} = {\text{ln}}\left( {2} \right)/\lambda , $$

where *λ* is the ^137^Cs loss rate in edible parts of the plants. The value of *λ* was obtained using the following equation:

3 $$ A_{{\text{t}}} = A_{0} \times {\text{ exp }}\left( { - \lambda \times t} \right), $$

where *A*_t_ is *T*_ag_ of ^137^Cs at time *t*, and *A*_0_ is estimated initial *T*_ag_. Temporal change in *T*_ag_ under a non-equilibrium condition is adequately described by a two-component exponential decline, comprising rapid and slow loss components^5^. In the present study, we adopted a single exponential equation in accordance with a previous study; Tagami and Uchida^10^ reported that the short effective half-life for three edible wild plant species of deciduous perennial spermatophytes was 103–230 days. Given that the present data were for the period 2012–2019, the effect of the rapid loss component was considered to be small. Parameter estimation in the single exponential model was conducted using the nls() function in R software 4.0.0^24^.

As the *T*_ag_ values showed a log-normal distribution for most species, Spearman’s rank correlation test was applied to examine the relationship between ^137^Cs deposition and concentration of ^137^Cs in the edible wild plants. One-way ANOVA and Tukey’s post hoc test on log-transformed *T*_ag_ values were used to evaluate the significance of differences among the species. These analyses were conducted using R software 4.0.0^24^. Significant differences were determined at a probability level of 0.05.

### Consent for publication

All authors have read and agreed to the published version of the manuscript.

## Results and discussion

### Comparison of *T*_ag_ calculated from publicly available data and actual measurement data

The calculated *T*_ag_ (m^2^/kg-FM) in each year is summarized for each species in Supplemental Table 1:

- The geometric means (GMs) of *T*_ag_ values calculated using the collected samples ranged from 8.1 × 10^−6^ to 2.5 × 10^−2^ m^2^/kg-FM; the minimum was for western bracken fern in 2019 and the maximum was for koshiabura in 2018 at Kawamata, Fukushima.
- The GMs of *T*_ag_ values calculated using the publicly available data ranged from 1.6 × 10^−5^ to 1.2 × 10^−2^ m^2^/kg-FM and thus were similar to the actual measurement data. The minimum GM was for udo in 2019 and the maximum was for koshiabura in 2019. The geometric standard deviation (GSD) range was 1.5–4.5.

**Figure 1:**
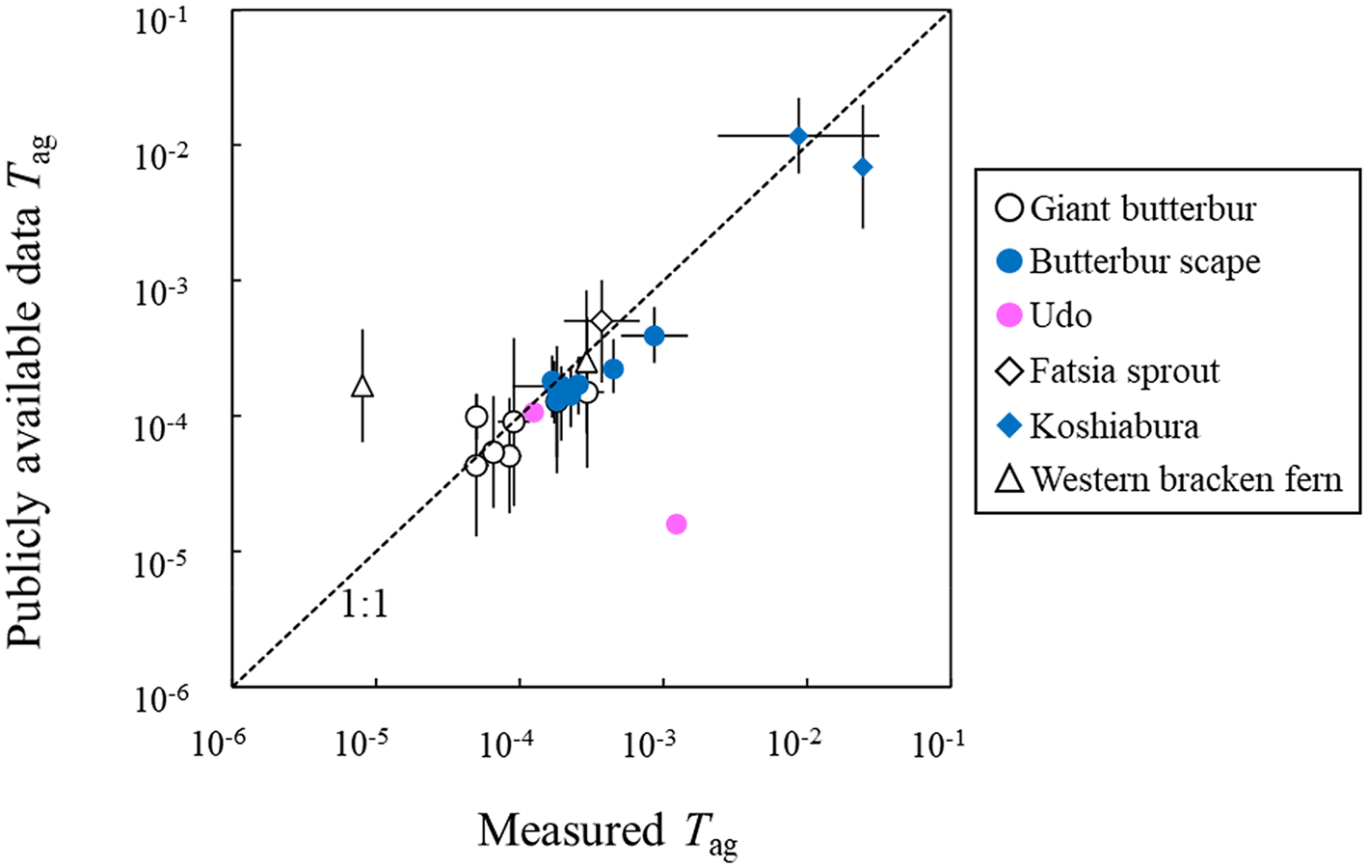
Comparison of annual geometric means of the aggregated transfer factor (*T*_ag_) calculated from publicly available data and actual measurement data. Circles, diamonds, and triangles indicate deciduous perennial spermatophytes, deciduous tree spermatophytes, and deciduous perennial pteridophytes, respectively. Values for individual years are represented by different points. Error bars indicate the geometric standard deviation in cases where more than three samples were available.

Annual GMs of *T*_ag_ values calculated from publicly available data and actual measurement data are compared in Fig. 1. The values for individual years are represented by different points. The *T*_ag_ values were distributed close to the 1:1 line, which suggested that *T*_ag_ values calculated from the publicly available data generally agreed with those calculated from actual measurements. Hence, an obvious overestimation of *T*_ag_ from the publicly available data described above was not observed in the present data. We confirmed that *T*_ag_ calculated from the publicly available food monitoring data and the total deposition data from the airborne survey are reliable surrogates for actual measurement samples. We discuss *T*_ag_ calculated from the publicly available data hereafter.

### Relationship between soil deposition and radioactivity in edible wild plants from publicly available data

**Figure 2:**
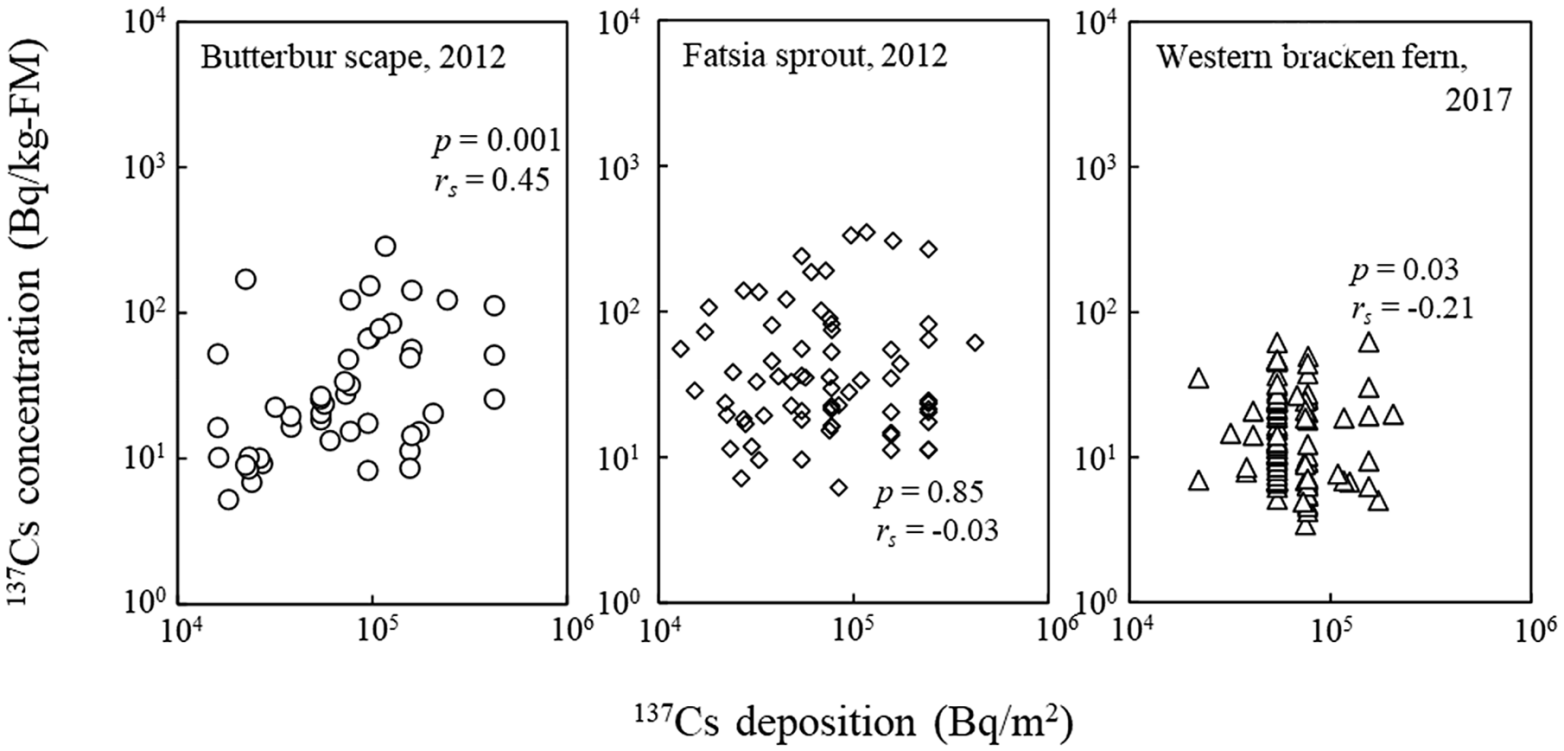
Correlation between deposition and concentration of ^137^Cs in three edible wild plants. Circles, diamonds, and triangles indicate butterbur scape, fatsia sprout, and western bracken fern, respectively. The three species are representative deciduous perennial and tree spermatophyte, and deciduous perennial pteridophyte, respectively, in the year of the maximum number of detections.

We confirmed the relationship between deposition and concentration of ^137^Cs for the publicly available data for butterbur scape, fatsia sprout, and western bracken fern in a year (Fig. 2), as a representative deciduous perennial and tree spermatophyte, and deciduous perennial pteridophyte, respectively, in the year of the maximum number of detections. Butterbur scape, fatsia sprout, and western bracken fern showed positive significant, nonsignificant, and weak negative significant correlations, respectively (Spearman’s rank correlation, butterbur scape, *p* = 0.001, *r*_*s*_ = 0.45; fatsia sprout, *p* = 0.85, *r*_*s*_ = − 0.03; western bracken fern, *p* = 0.03, *r*_*s*_ = − 0.21). Among 29 subdata with more than 20 detections for each species in a year, in addition to the data shown in Fig. 2, 13 showed statistically significant positive correlations (Butterbur scape in 2014 and 2016; bamboo shoot in 2012, and 2014 − 2019; fatsia sprout in 2013 and 2016; koshiabura in 2013; and ostrich fern in 2012), and western bracken fern in 2017 showed a significant negative correlation. These weak correlations may be affected by uncertainty in the deposition data. We used a representative deposition value for each municipality and the original deposition data grid was of low resolution (see the “Methods” section Radiocesium deposition data from airborne survey). Especially for the cases lacking a clear positive correlation, the degree of radiocesium absorption by edible wild plants was largely different even in the same deposition. Radiocesium uptake by plants in an environment is also affected by other factors (e.g., soil characteristics^25,26^). The edible wild plants targeted in the present study were not cultivated but were collected in a variety of environments, such as forests with high organic matter content in the soil and paddy field margins with poorly drained soil high in clay content, although we cannot precisely confirm the growth environment of each species included in the present study.

### Temporal change in *T*_ag_

**Figure 3:**
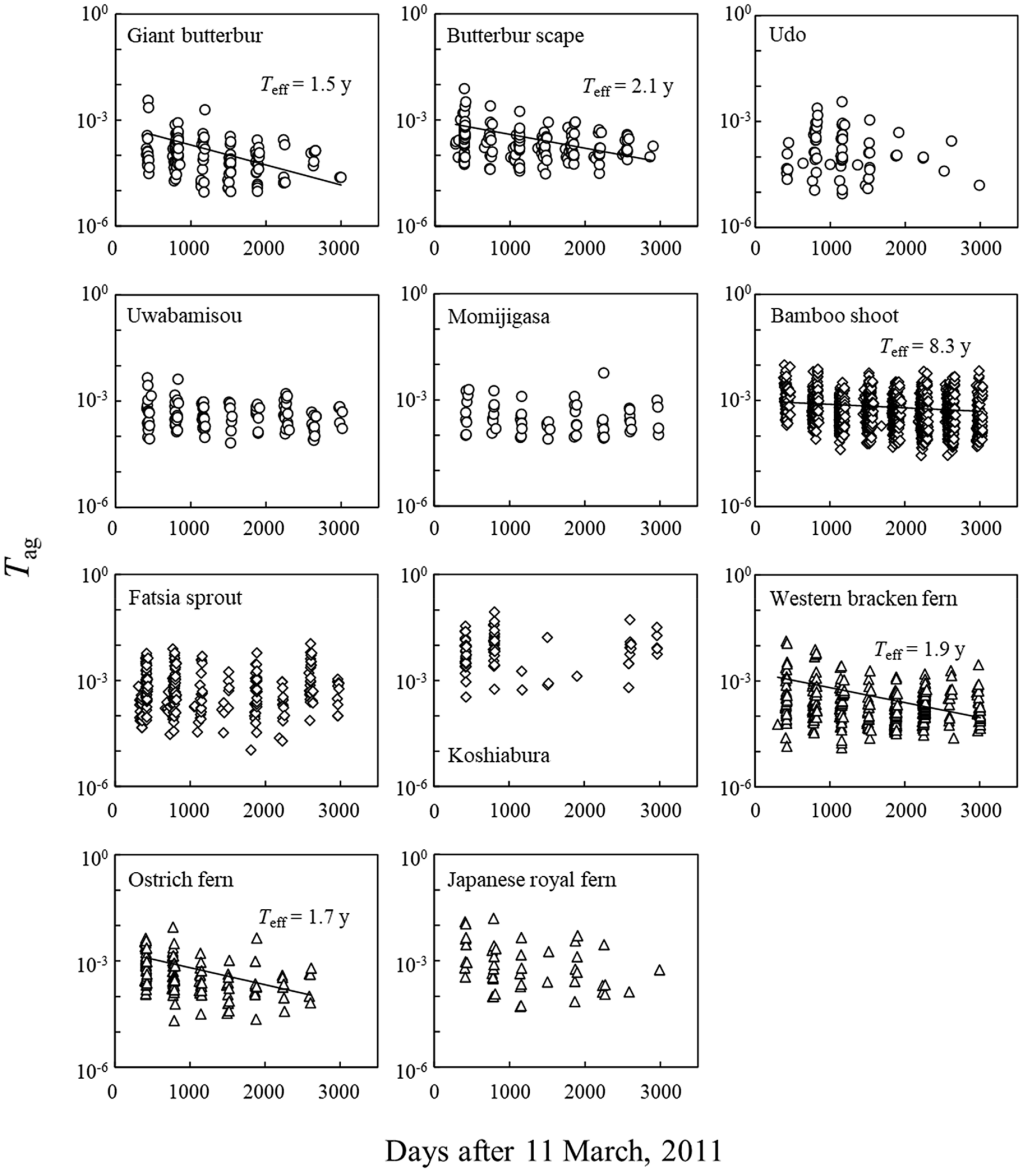
Temporal change in the aggregated transfer factor (*T*_ag_) in the period 2012–2019. Circles, diamonds, and triangles indicate deciduous perennial spermatophytes, deciduous tree spermatophytes (including bamboo shoot), and deciduous perennial pteridophytes, respectively. Single exponential fitted lines are shown. Solid lines indicate statistically significant parameters (see Table 2).

The time-dependence of *T*_ag_ for each species in the period 2012–2019 is shown in Fig. 3. The *T*_ag_ values of deciduous perennial spermatophytes and pteridophytes showed a decreasing trend with time. Given that the bioavailability of ^137^Cs in the soil in the plant root zone decreased with time, as observed in previous studies^27,28^, we also observed a decrease in *T*_ag_. The *T*_ag_ of deciduous trees did not show a decreasing trend with time.

After the Chernobyl nuclear accident, radiocesium concentrations in deciduous tree leaves decreased with time owing to the effect of direct deposition at an early stage and the following root uptake effect^29^, and the *T*_ag_ of tree leaves decreased accordingly. In previous studies conducted in orchards after the Chernobyl and Fukushima accidents, radiocesium concentrations in deciduous tree leaves showed a decreasing trend^30,31^. The lack of a declining trend for woody edible wild plants *T*_ag_ in the present study may be due to a smaller effect of direct deposition at the early stage resulting from interception by tall tree canopies in the vicinity. The height of trees with edible wild plants is usually at eye level. The samples collected soon after the accident were possibly affected by direct deposition, whereas in the latter study period, many of the data were from trees grown after the accident. If the effect of direct deposition was large, a declining trend in *T*_ag_ might have been observed as observed in orchards. Thus, the absence of a declining trend in *T*_ag_ indicates that the effect of direct deposition was relatively small.

As an additional possibility for the absence of a declining trend in tree *T*_ag_, the continuous supply of bioavailable radiocesium from the organic layer on the forest floor may affect the temporal change in *T*_ag_. Compared with the managed conditions in orchards of previous studies^30,31^, an organic layer develops on the soil surface in a forest and, therefore, reabsorption of radiocesium from the organic layer via the roots may be more active. Imamura et al.^17^ also observed a similar trend to that in the present study, namely that radiocesium concentrations in leaves of the canopies of the deciduous tree konara oak (*Quercus serrata*) did not show a temporal change from 2011 to 2015 in two Fukushima forests. These authors’ results included the effect of direct deposition on the tree bodies at an early stage of the accident, although the emergence of leaves was after the deposition. Nevertheless, a clear decreasing trend in the radiocesium concentration was not observed, which implies that a deciduous tree actively absorbs radiocesium via the roots in Fukushima forests, and a sufficient amount of radiocesium is absorbed to conceal a decline at an early stage owing to the effect of direct deposition.

Single exponential fitted lines for each species are shown in Fig. 3. The estimated parameters and the *T*_eff_ (year) calculated with Eq. (2) in “Methods” section are presented in Table 2. The *T*_eff_ for *T*_ag_ values that showed a decreasing trend was approximately 2 years, except for bamboo shoot. Tagami and Uchida^10^ reported that the *T*_eff_ of the slow loss component for three edible wild plants of deciduous perennial spermatophytes was 970–3830 days. The ^137^Cs decline in pteridophytes, and deciduous shrub and herbaceous species on the floor of European forests was reported to be 1.2–8 years for *T*_eff_ excluding the rapid loss component after the Chernobyl nuclear accident^32^. The present results are thus within the range of previous studies.

**Table 2:** Estimated parameters and standard errors for correlations of *T*_ag_ (m^2^/kg-FM) in the period 2012–2019 with time (day) calculated using Eq. (3) and effective half-lives [*T*_eff_, (year)] calculated using Eq. (2) for 11 parts of 10 edible wild plant species. *A*_0_ is estimated initial *T*_ag_, and *λ* (/day) is the ^137^Cs loss rate in edible parts of the plants.

Vernacular name	*A*_0_ (m^2^/kg-FM)				*λ* (/day)				*T*_eff_ (year)^a^		
Giant butterbur	7.27 × 10^−4^	±	2.95 × 10^−4^	*	1.31 × 10^−3^	±	5.31 × 10^−4^	*	1.5	±	0.6
Butterbur scape	9.59 × 10^−4^	±	2.19 × 10^−4^	***	8.97 × 10^−4^	±	3.12 × 10^−4^	**	2.1	±	0.7
Udo	4.28 × 10^−4^	±	2.07 × 10^−4^	*	2.06 × 10^−4^	±	4.24 × 10^−4^				
Uwabamisou	8.73 × 10^−4^	±	1.83 × 10^−4^	***	2.99 × 10^−4^	±	1.56 × 10^−4^	†	(6.4	±	3.3)^b^
Momijigasa	6.43 × 10^−4^	±	2.79 × 10^−4^	*	1.31 × 10^−4^	±	2.68 × 10^−4^				
Bamboo shoot	9.80 × 10^−4^	±	6.28 × 10^−5^	***	2.27 × 10^−4^	±	3.84 × 10^−5^	***	8.3	±	1.4
Fatsia sprout	3.74 × 10^−4^	±	1.81 × 10^−4^	†	− 1.14 × 10^−4^	±	2.40 × 10^−4^				
Koshiabura	1.16 × 10^−2^	±	2.45 × 10^−3^	***	− 3.06 × 10^−5^	±	1.47 × 10^−4^				
Western bracken fern	1.74 × 10^−3^	±	3.26 × 10^−4^	***	9.75 × 10^−4^	±	1.93 × 10^−4^	***	1.9	±	0.4
Ostrich fern	1.97 × 10^−3^	±	5.23 × 10^−4^	***	1.11 × 10^−3^	±	4.02 × 10^−4^	**	1.7	±	0.6
Japanese royal fern	7.20 × 10^−3^	±	3.72 × 10^−3^	†	1.48 × 10^−3^	±	7.91 × 10^−4^	†	(1.3	±	0.7)^b^

***p < 0.001, **p < 0.01, *p < 0.05, †*p* < 0.1.

^a^*T*_eff_ was calculated only when *λ* was statistically significant.

^b^Rough estimation values with nonsignificant (0.05 < *p* < 0.1) parameters.

For bamboo shoot, applying a single exponential function, a relatively long *T*_eff_ of 8.3 years was estimated. The *T*_ag_ decreased between 2012 and 2014, and thereafter no notable change was observed. This observation may reflect the effect of rapid and a slow loss components. Indeed, we applied a two-component exponential function for bamboo shoot, and observed *T*_eff_ of 0.7 years and − 7.8 years for the rapid and slow loss components, respectively. For edible wild tree species, statistically significant single exponential fitted lines were not observed, which reflected the absence of change in *T*_ag_ with time, as discussed above in this section.

The *T*_ag_ varied for all species, varying by 1–3 orders of magnitude within a year that included more than two detections (Fig. 3, Supplemental Table 1). As demonstrated in previous studies^5^, the present study also showed substantial variation in *T*_ag_ values, which may be for several reasons. Recently, Tagami et al.^12^ calculated *T*_ag_ using the radiocesium concentration in edible wild plants measured by local municipalities from higher-resolution publicly available data (accurate to district level) for giant butterbur, bamboo shoot, fatsia sprout, and koshiabura. The municipalities in these authors’ study are located within the present study area. These authors’ results differed in being one or two orders of magnitude smaller than the present results. The lower resolution of the present deposition data may be one of the causes of the greater *T*_ag_ variation. The other source of variation is the site dependency of radiocesium absorption by edible wild plants from the soil as described above. Clarification of factors that contribute to the variation in *T*_ag_ other than ^137^Cs deposition, and its trends consistent with species, is necessary, which will decrease uncertainty and lead to more accurate estimation of *T*_ag_ of ^137^Cs with wild plants.

### Summary of *T*_ag_ for estimation of long-term ingestion dose to the public

To estimate long-term potential ingestion dose to the public, *T*_ag_ with small temporal variability excluding high values at the early stage after the accident is required. However, for the edible wild plant species in the present study, no *T*_ag_ information in an equilibrium condition from before the Fukushima accident is available. Therefore, average values of *T*_ag_ for the period after the decrease in *T*_ag_ has weakened and a certain number of samples is available would be appropriate. The *T*_eff_ for *T*_ag_ showing a decreasing trend was approximately 2 years except for bamboo shoot, which has not shown any temporal variation since 2014. The *T*_ag_ for the other species, udo, uwabamisou, momijigasa, fatsia sprout, koshiabura and Japanese royal fern, has not shown temporal variation throughout 2012–2019 (see the “Results and discussion” section Temporal change in *T*_ag_). Therefore, *T*_ag_ values since 2014 are applicable for estimation of long-term potential ingestion dose to the public. The GMs and GSDs of the *T*_ag_ values for 2014–2019 for each species are shown in Table 3 listed in order of decreasing GM.

**Table 3:** Aggregated transfer factor (m^2^/kg-FM) calculated from publicly available data for 2014–2019 for 11 parts of 10 edible wild plant species.

Vernacular name	Plant category	*T*_ag_ (m^2^/kg-FM)						Comparison with previous studies
		*N*	GM*		GSD	Minimum	Maximum	
Koshiabura	Spermatophyte, deciduous tree	22	5.2 × 10^−3^	a	3.5	5.4 × 10^−4^	5.3 × 10^−2^	7.3 × 10^−3^,^12^
								7.2 × 10^−2^ on a dry-weight basis^33^
Fatsia sprout	Spermatophyte, deciduous tree	115	4.3 × 10^−4^	b	4.0	1.1 × 10^−5^	1.1 × 10^−2^	1.1 × 10^−3^,^12^
Japanese royal fern	Pteridophyte, deciduous perennial	24	4.2 × 10^−4^	bc	3.9	5.1 × 10^−5^	5.0 × 10^−3^	1.4 × 10^−4^–7.0 × 10^−4^ for spermatophyte and pteridophyte perennials^10^
Bamboo shoot	Spermatophyte	2170	3.9 × 10^−4^	b	2.5	2.8 × 10^−5^	6.9 × 10^−3^	5.1 × 10^−4^,^12^
Uwabamisou	Spermatophyte, deciduous perennial	69	3.6 × 10^−4^	b	2.1	6.6 × 10^−5^	1.6 × 10^−3^	5.3 × 10^−5^–1.6 × 10^−4^ for deciduous spermatophyte perennials^10^
Momijigasa	Spermatophyte, deciduous perennial	40	2.5 × 10^−4^	bcd	2.5	7.8 × 10^−5^	5.8 × 10^−3^	1.4 × 10^−4^–7.0 × 10^−4^ for spermatophyte and pteridophyte perennials^10^
Ostrich fern	Pteridophyte, deciduous perennial	52	2.0 × 10^−4^	cde	2.8	2.3 × 10^−5^	4.4 × 10^−3^	1.4 × 10^−4^–7.0 × 10^−4^ for spermatophyte and pteridophyte perennials^10^
Western bracken fern	Pteridophyte, deciduous perennial	329	1.9 × 10^−4^	de	3.3	1.3 × 10^−5^	2.7 × 10^−3^	
Butterbur scape	Spermatophyte, deciduous perennial	96	1.7 × 10^−4^	de	2.2	3.0 × 10^−5^	1.7 × 10^−3^	5.3 × 10^−5^–1.6 × 10^−4^ for deciduous spermatophyte perennials^10^
Udo	Spermatophyte, deciduous perennial	40	1.1 × 10^−4^	e	4.0	9.0 × 10^−6^	3.7 × 10^−3^	1.4 × 10^−4^–7.0 × 10^−4^ for spermatophyte and pteridophyte perennials^10^
Giant butterbur	Spermatophyte, deciduous perennial	69	6.1 × 10^−5^	f	3.1	9.2 × 10^−6^	1.9 × 10^−3^	1.4 × 10^−4^, ^12^
								5.3 × 10^−5^–1.6 × 10^−4^ for deciduous spermatophyte perennials^10^
								1.4 × 10^−4^–7.0 × 10^−4^ for spermatophyte and pteridophyte perennials^10^

*T*_ag_, Aggregated transfer factor; GM, Geometric mean; GSD, geometric standard deviation.

*Different lower-case letters indicate a significant difference (one-way ANOVA with Tukey’s post hoc test, *p* < 0.05).

Significant differences in *T*_ag_ were observed among the species (one-way ANOVA with Tukey’s post hoc test, *p* < 0.05). The maximum GM was 5.2 × 10^−3^ m^2^/kg-FM for koshiabura. The second-highest GM was that observed for fatsia sprout (4.3 × 10^−4^ m^2^/kg-FM). The tree species showed higher *T*_ag_ values than those of the five spermatophyte perennials (6.1 × 10^−5^ to 3.6 × 10^−4^ m^2^/kg-FM). The *T*_ag_ for bamboo shoot, which shows characteristics of tree species and herbaceous plants, was intermediate (3.9 × 10^−4^ m^2^/kg-FM) between those two plant categories. The *T*_ag_ values for pteridophytes ranged from 1.9 × 10^−4^ to 4.3 × 10^−4^ m^2^/kg-FM, and thus were similar or slightly higher than those of spermatophyte perennials. These results implied that the *T*_ag_ of edible wild plants showed certain trends consistent with the plant category.

Similar *T*_ag_ values have been reported in previous studies (Table 3). Tagami et al.^12^ reported *T*_ag_ values for the tree species koshiabura and fatsia sprout of 7.3 × 10^−3^ and 1.1 × 10^−3^ m^2^/kg-FM, respectively, and values of 5.1 × 10^−4^ and 1.4 × 10^−4^ m^2^/kg-FM for the spermatophyte deciduous perennials bamboo shoot and giant butterbur, respectively. Kiyono et al.^33^ reported *T*_ag_ for koshiabura on a dry-weight basis of 7.2 × 10^−2^ m^2^/kg, which was similar to the present result adjusted for the water content of the actual measured samples of 86%–88%. Tagami and Uchida^10^ calculated that the *T*_ag_ of three species of deciduous spermatophyte perennials ranged from 5.3 × 10^−5^ to 1.6 × 10^−4^ m^2^/kg-FM, and that for five species of spermatophyte and pteridophyte perennials ranged from 1.4 × 10^−4^ to 7.0 × 10^−4^ m^2^/kg-FM. The exceptionally high ^137^Cs concentration in koshiabura has been well documented in the Fukushima region^34,35^. Several factors have been proposed: the shallow root zone of koshiabura corresponds to a surface layer of forest soil containing a high concentration of bioavailable radiocesium, and the root endophytic bacteria of koshiabura make radiocesium in the soil easier to absorb for koshiabura^36,37^. The *T*_ag_ of koshiabura is an order of magnitude higher than that of most other species studied (and two orders of magnitude for giant butterbur). Therefore, consumption of koshiabura contributes to a higher potential ingestion dose compared with those of the other species.

No data are available for *T*_ag_ evaluated before the Fukushima accident for the present study species. Therefore, we cannot verify whether the obtained *T*_ag_ values are in the range under an equilibrium condition. Somewhat informative data have been recorded by the Nuclear Regulation Authority, and include measurements of ^137^Cs radioactivity in mugwort (*Artemisia indica* Willd.) and soil collected before the accident in Miyagi Prefecture, which is in the same region as Fukushima^38^. Mugwort is an edible deciduous spermatophyte perennial of the Asteraceae that grows in fields or mountains, but was not included in the present study. The giant butterbur, butterbur scape, and momijigasa are also deciduous spermatophyte perennials of the Asteraceae. The ^137^Cs activities in 2001–2010 for mugwort and soil were 0.029–0.16 Bq/kg-FM and 108–1455 Bq/m^2^, respectively (decay-corrected to 1 January, 2001). The *T*_ag_ values derived from the measurement data are generally at a level consistent with these three species in Table 3. The *T*_ag_ values summarized in 2014–2019 are presumed to be close to those observed under an equilibrium condition, and are suggested to be useful to estimate the long-term potential ingestion dose to the public resulting from consumption of these edible wild plants.

## Conclusion

We calculated *T*_ag_ for 11 parts of 10 common edible wild plants using publicly available data for 2012–2019. Each species shows variation in *T*_ag_ of about two orders of magnitude in 1 year. The reason may partly be because the sampling locations were identified only at the municipal level and uncertainty in the deposition data. Several species showed trends for decline in *T*_ag_ and the trends have become relatively weaker since 2014. Variation in *T*_ag_ of each species was substantial, but averaged *T*_ag_ values for each species since 2014 are applicable for estimation of long-term potential ingestion dose to the public. The averaged *T*_ag_ ranged over approximately two orders of magnitude among the 10 species, and to a certain extent trends were consistent with plant category. Tree species tend to show a higher *T*_ag_ than spermatophyte and pteridophyte perennials, and bamboo shoot *T*_ag_ is intermediate between that of trees and spermatophyte perennials. Clarification of the factors underlying this variation is necessary, which will lead to accurate *T*_ag_ calculation.

## Supplementary Information

Supplementary Information.

## Data Availability

The data that support the findings of this study are available from the corresponding author upon reasonable request.

## Additional information

We comply with relevant guidelines and legislation regarding the sample collection in the present study. The plant species in the study are not endangered. The edible wild plant samples, udo, fatsia sprout, koshiabura, and western bracken fern in 2018–2019 collected in Kawamata town and Iitate village, were collected by local residents as part of their wild foods collection activities, and we do not own the measurement samples. The samples of giant butterbur and butterbur scape in 2012–2019 collected on the campus of QST in Chiba Prefecture are stored in QST. Voucher specimens of plant materials in the present study do not exist.
